# Pollution of Lakes and Rivers

**Published:** 1913-01

**Authors:** Chas. A. Hodgetts

**Affiliations:** Medical Advisor, The Commission of Conservation


### BUFFALO MEDICAL JOURNAL

VOL. LXVIII JANUARY, 1913 No. 6

### ORIGINAL ARTICLES

### Pollution of Lakes and Rivers

**From a Canadian Viewpoint**

By CHAS. A. HODGETTS, M.D., D.P.H., L.R.C.P., Lon., F.R.San-I.
Medical Advisor, The Commission of Conservation

IT is quite apparent, even to a most casual student of the question, that while the great tide of industrial development has brought to us wealth, both national and personal, yet that same tide has brought with it sickness, suffering and death, which count for national loss and personal suffering. Man, the manufacturer, in his haste to get rich quick, has transgressed the laws of health: it is safe to say that there is not a river on the Continent of North America which is not an open sewer; and our fair lakes are defiled by the overt acts of men and women everywhere.

The stigma which rests upon our so-called western civilization, is the utter indifference to the value of human life which we find in evidence everywhere. We are now in the thraldom of a white slavery, one manifestation of which is the utter disregard we have shown for the health of our people by the indiscriminate pollution of our lakes, rivers and streams. We wantonly defile one of the greatest blessings with which a Great Creator has blessed us—for travel anywhere throughout the world and you will not find anything to compare with our great natural reservoirs of once pure and limpid waters, which now are the fountains from which flow disease and death.

It is a fact that typhoid fever, which is a water-borne disease, as well as many intestinal ailments, of which the sanitarian has no definite statistical data, are wide spread. Certainly typhoid is much more common in America than in the crowded countries of Europe. We have only to turn to the statistics of the mortality of typhoid fever to learn the truth of this statement. The following will serve to indicate the relative conditions as to the mortality of this one disease in some of the principal cities of the United States, Canada and Europe:

TABLE B.—TYPHOID FEVER.

Cities of the United States, bordering Great Lakes, mortality rate per 100,000 of population, 1900-1908, (inclusive).

| CITIES | RATE PER 100,000 OF POPULATION BY YEARS. | | | | | | | | |
|---|---|---|---|---|---|---|---|---|---|
| | 1900 | 1901 | 1902 | 1903 | 1904 | 1905 | 1906 | 1907 | 1908 |
| Ashtabula ........ | 7.7 | 44.9 | 36.3 | 49.4 | 137.1 | 60.0 | 38.9 | 19.0 | 86.2 |
| Buffalo .......... | 23.5 | 27.1 | 33.7 | 34.6 | 24.2 | 24.4 | 23.6 | 29.2 | 20.7 |
| Chicago ........ | 21.1 | 29.8 | 45.1 | 32.1 | 20.2 | 16.5 | 18.3 | 17.7 | 15.3 |
| Cleveland ........ | 56.8 | 34.9 | 35.5 | 115.0 | 49.6 | 14.9 | 20.2 | 18.9 | 12.6 |
| Detroit .......... | 28.4 | 20.1 | 23.5 | 20.0 | 17.6 | 21.2 | 22.3 | 28.3 | 22.3 |
| Duluth ........... | 109.5 | 74.1 | 53.7 | 64.8 | 54.4 | 44.7 | 46.0 | 41.6 | 56.8 |
| Milwaukee ....... | 19.3 | 22.1 | 15.1 | 16.8 | 13.6 | 22.7 | 30.5 | 25.7 | 17.4 |
| Niagara Falls .... | 107.9 | 143.9 | 130.4 | 126.9 | 139.8 | 181.6 | 147.3 | 126.8 | 98. |
| Ogdensburg ....... | 55.4 | 20.4 | 95.0 | 54.2 | 60.9 | 40.5 | 87.6 | 40.4 | 33.6 |
| Port Huron ...... | 47.0 | 41.3 | 61.2 | 25.2 | 34.9 | 14.8 | 53.8 | 43.5 | 19.1 |
| Sault Ste. Marie.. | 132.9 | 92.9 | 172.9 | 115.9 | 52.4 | 68.6 | 58.9 | 16.5 | 72.9 |
| Toledo ........... | 41.0 | 32.2 | 34.7 | 29.5 | 37.2 | 45.7 | 45.0 | 36.4 | 40.1 |

TABLE C.—TYPHOID FEVER.

Death rate of nine countries, per 100,00 of population.

| YEAR GROUP | COUNTRIES | RATE PER 100,000 OF POPULATION |
|---|---|---|
| 1901-1905 ........ | Scotland ........ | 6.2 |
| | Germany ....... | 7.6 |
| | Eng. and Wales.. | 11.2 |
| | Belgium ......... | 16.8 |
| 1901,1904 ........ | Austria .......... | 19.9 |
| | Hungary ......... | 28.3 |
| | Italy ............ | 35.2 |
| 1901 (census) ... | Canada .......... | 35.5 |
| 1901-1904 ........ | United States .... | 46.0 (estimated) |

Fortunately the cry has gone forth from the suffering public: " 'What must we do to be saved' from this preventable scourge?" In some cases it has found a sympathetic response from the Sanitary authorities, but, up to the present time, it has not been heard or else it has not pierced the hearts of our so-called statesmen who are too intent on political exigencies to pay any attention to that which is the very strength of the nation, viz., the men and women of today and the children whose lives should be conserved for the sake of our future prosperity. The most of our law makers are more concerned about the raising and breeding of good cattle and the extension of manufactures than they are about this vital question. They are not sensible enough of the fact that the conservation of the lives of our people is of infinitely greater importance.

In some quarters legislators, even in this western hemisphere, have demonstrated the fact that statesmen are not dead—only lost amongst the mass of political briars and weeds which incumber our legislative halls. No less than four of the provincial governments of Canada have recently placed upon their statute books laws which will prevent the pollution of Canadian rivers and lakes by raw sewage and factory waste. In these provinces the truth has been recognized that all bodies of fresh water were given for man's use, not for his abuse. These laws will not produce an immediate change in the present unsatisfactory sanitary conditions, but sooner or later the effects will be noticeable and the results from a hygenic as well as from an aesthetic standpoint, will be salutary.

Of course, it cannot be expected that were every town and city to treat their sewage effluent so as to render it non non-putrescible and non-pathogenetic there would be assured to all consumers of lake and river waters supplies that would be guaranteed pure. It can be stated, however, that with the removal of all gross material from sewage and its subsequent partial purification and sterilization, the much vaunted grossly abused, so-called "'natural purification' by dilution method" will give infinitely better results. At present, the claim that an all-wise Province will do all the work thrust upon Him by engineers and municipal authorities, and, also, by some sanitarians, is a blasphemy as great as civilized (?) man ever perpetrated. For it is safe to say that this failure of the dilution method is clearly demonstrated in the sixty million dollar experiment of the Chicago Drainage Commission, which has, to be sure, succeeded in developing power but certainly has miserably failed in demonstrating that even by using as much water as flows over the American falls at Niagara, their sewage and factory waste has been disposed of either efficiently or in a sanitary manner. There is no question that the system adopted has minimized the danger of the pollution of the city water supply, but, it cannot be said that it has solved the more difficult one: of how to dispose of the sewage and factory waste of that city.

In Canada, sanitary authorities have been directing their attention to the prevention of the pollution of waterways and their tributaries. At the present time, four of the provinces of the Dominion have made it impossible for municipalities to continue the defiling of our rivers and lakes by making them the receptacles of untreated sewage. Notwithstanding our small population and vast extent of territory, we realize the necessity thus early in our national history, of protecting the lives of our people and conserving the beauty of our lakes and rivers. The fact that the legislatures of Ontario, Manitoba, Saskatchewan and Alberta have placed upon the statute book, laws along the lines just indicated, is proof that the fact has already been brought home to the people of these important provinces, that "nature" does not dispose of raw rewage when the dilution method is adopted.

Where international waterways are concerned, the problem is a complex one. The chief offenders are naturally the more numerous towns and cities to the south of the boundary line, although it is simply a difference in degree as regards the quantity of pollution, nothing more; for the citizens of both Canada and the United States are guilty. We, in Canada, however, have taken the stand that municipalities must provide for the treatment of their sewage, thereby lessening the contamination of our pure water lakes and rivers. Health authorities in Canada are unanimous on the question of the prevention of pollution, not only because they hope to secure for municipal authorities pure water reservoirs from which they may obtain palatable waters, but because they believe that municipalities in their own interests should not continue to make these reservoirs the receptacle for raw and untreated sewage, thus wantonly making them the sources of disease and death to many thousands who either innocently or compulsorily use the same.

We firmly believe in Canada that it is a cardinal principle to be observed by all citizens that sewage should be properly and scientifically treated and disposed of; that no one should discharge into any body of water a sewage effluent that is not non-putrescrible and non-pathogenic. We believe that this is sound in principle and possible in practice. Further, we believe that, unless municipalities will secure their water supplies from carefully protected upland sources, it will be necessary in most instances to filter the water.

As regards the disposal of sewage by lake and river steamers, we believe that the most stringent rules should be adopted and rigidly enforced by the proper authorities to prevent the indiscriminate pollution of these waters. At the same time, we believe that the water supply should be taken only from known sources that are certified to be pure.

The only way these international waters can be safeguarded and the public protected, is by a co-ordinated movement of our Federal and State or Provincial authorities, and we feel sure that it is the wish of every right thinking citizen of both countries that this great sanitary movement may be successful.

---

DESTRUCTION OF INTESTINAL ANAEROBES BY OXYGEN. Adolf Schmidt, *Inter-state Med. Jour.*, July, 1912, introduces oxygen by duodenal tube, once or twice daily. 4 liters or more can be introduced without undue distention.

